# Suv39h1: Targeting epigenetics to treat liver fibrosis

**DOI:** 10.1111/jcmm.18479

**Published:** 2024-05-31

**Authors:** Kostas A. Papavassiliou, Athanasios G. Papavassiliou

**Affiliations:** ^1^ First University Department of Respiratory Medicine ‘Sotiria’ Hospital, Medical School, National and Kapodistrian University of Athens Athens Greece; ^2^ Department of Biological Chemistry Medical School, National and Kapodistrian University of Athens Athens Greece

A recent study published in *Gut* by Kong et al. revealed that a suppressor of variegation 3–9 homologue 1 (Suv39h1)‐driven epigenetic mechanism is involved in the regulation of hepatic stellate cell (HSC) to myofibroblast transition, resulting in liver fibrosis (Figure 1A).^1^ Remarkably, the authors also found that Suv39h1 can be successfully inhibited with a small‐molecule natural product isolated from Chaetomium fungi belonging to thiodiketopyrazine complexes, namely chaetocin, leading to the attenuation of liver fibrosis.

**FIGURE 1:**
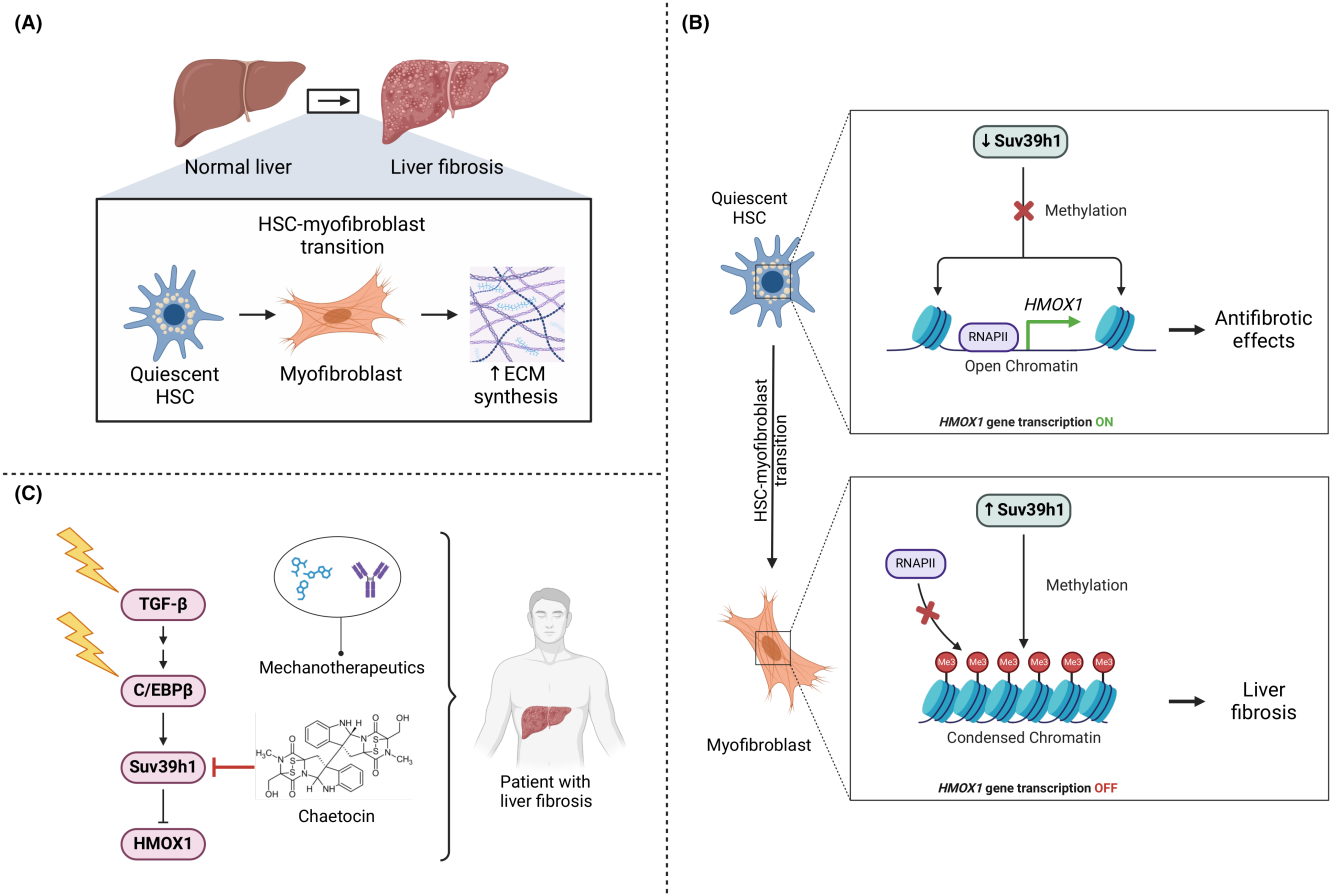
Suv39h1 is a key regulator of HSC‐myofibroblast transition, which promotes liver fibrosis, and can be targeted with the small‐molecule compound chaetocin. (A) In liver fibrosis, quiescent HSCs transdifferentiate into ECM‐secreting myofibroblasts. (B) Downregulated Suv39h1 in quiescent HSCs is not able to methylate histones and, thus, repress the gene expression of HMOX1, allowing the latter to freely mediate its antifibrotic effects, whereas upregulated Suv39h1 during HSC‐myofibroblast transition and in myofibroblasts functions to trimethylate histones (Me3) and, thus, repress the gene expression of HMOX1, not allowing the latter to mediate its antifibrotic effects, leading to liver fibrosis. (C) Signalling pathway involved in HSC‐myofibroblast transition fostering liver fibrosis and its targetable components, including Suv39h1, C/EBPβ and TGF‐β. Future therapeutic strategies against liver fibrosis may be based on combinatorial drug regimens, comprising Suv39h1 inhibitors with C/EBPβ and/or Suv39h1‐recruited transcription factor inhibitors, TGF‐β inhibitors or mechanotherapeutics that target the mechanobiological aspects (i.e. ECM, components of mechanotransduction signalling pathways) related to liver fibrosis. Thunder signs represent targeting. C/EBPβ, CCAAT enhancer binding protein beta; ECM, extracellular matrix; HMOX1, heme oxygenase 1; HSC, hepatic stellate cell; Me3, histone trimethylation; RNAPII, RNA polymerase II; Suv39h1, suppressor of variegation 3–9 homologue 1; TGF‐β, transforming growth factor beta. This figure was created based on the tools provided by Biorender.com (https://biorender.com/).

Liver fibrosis represents a dynamic pathological process characterized by immune cell infiltration and inflammation, as well as the aberrant deposition of excess extracellular matrix (ECM) components, which are secreted by liver myofibroblasts, within the liver tissue as result of a continuous reparative response to a plethora of injurious stimuli. This ever‐going process leads to accumulating abnormalities in liver architecture and function, progressing to end‐stage liver diseases, such as cirrhosis and hepatocellular carcinoma (HCC).^2^ Multiple studies suggest that liver myofibroblasts are mainly derived from activated HSCs and that the HSC to fibrogenic myofibroblast transition is the rate‐limiting step in liver fibrosis.^3^, ^4^, ^5^ With this in mind and given that there is a lack of therapeutic interventions for liver fibrosis, focusing on the HSC‐myofibroblast transition as a target and developing strategies to block this transition may generate the most promising treatments in patients with fibrotic livers.

Interestingly, Kong et al. uncovered an important and targetable signalling axis in HSC‐myofibroblast transition, which entails transforming growth factor beta (TGF‐β), CCAAT enhancer binding protein beta (C/EBPβ), Suv39h1 and heme oxygenase 1 (HMOX1). In a nutshell, the authors' data indicate that when a quiescent HSC becomes stimulated by pro‐fibrogenic growth factors, such as TGF‐β, downstream intracellular signalling causes C/EBPβ to induce the gene expression of Suv39h1, which in turn downregulates, in an epigenetic manner via histone 3 lysine 9 trimethylation (H3K9me3), the gene expression of HMOX1, not allowing the latter to mediate its antifibrotic effects and, thus, promoting liver fibrosis (Figure 1B). The translational potential of this work by Kong et al. is based on the finding showing that administration of a small‐molecule compound, chaetocin, was able to potently inhibit Suv39h1 and successfully mitigate liver fibrosis in a relevant mouse model.

However, as the authors note, there are some limitations with this work that hinder the translation of its findings into clinical practice. For example, it was observed that pharmacological inhibition of Suv39h1 by chaetocin differed from genetic knockdown of *Suv39h1* in terms of transcriptomic changes, hinting to potential off‐target effects of the drug. Notwithstanding, while in HSC‐ and myofibroblast‐specific *Suv39h1* knockout mice there was no significant difference with respect to liver injury and inflammation when compared to control, in chaetocin‐treated mice, both liver injury and inflammation were reduced. Therefore, chaetocin appears to not only dampen liver fibrogenesis, but also liver injury and inflammation, effects that are beneficial in the setting of liver fibrosis. Perhaps, it would be worth investigating the molecular underpinnings of these ‘positive off‐target effects’ of chaetocin to gain a better understanding of its mechanism of action.

Regarding the therapeutic potential of targeting Suv39h1 in patients with liver fibrosis, further research is needed to identify and validate new generation Suv39h1 inhibitors that are more target‐selective and less toxic. Along these lines, a selective Suv39h1 inhibitor, ETP69 (a chaetocin A analog), and its successful application in treating memory loss in aged rodents has been reported.^6^ Additionally, two other recent studies on colon cancer used the chemically synthesized small‐molecule compound F5446 to more potently and selectively inhibit Suv39h1, resulting in enhancement of effector gene expression in tumour‐infiltrating cytotoxic T lymphocytes to suppress colon tumour growth, and suppressing human colon tumour xenograft growth in vivo, respectively.^7^, ^8^ Exploring these Suv39h1 inhibitors for the intervention of liver fibrosis would be intriguing. Finally, another therapeutic aspect that demands further probing in liver fibrosis is the combination of Suv39h1 inhibitors with other epigenetically acting drugs (e.g. C/EBPβ inhibitors), small‐molecule compounds disrupting Suv39h1‐gene‐specific transcription factor interactions, TGF‐β targeting agents or mechanotherapeutics that target the mechanobiological features (i.e. ECM, mechanotransduction signalling cascades) of liver fibrosis (Figure 1C).^9^, ^10^, ^11^, ^12^

Future investigations should augment our multidisciplinary efforts by fusing data from omics technologies (genomics, epigenomics, transcriptomics, proteomics, metabolomics) with advances in computer‐aided drug design (computational chemistry, molecular modelling) methods and drug delivery systems (micelles, nanoparticles). Only then will we have an opportunity to combat the intricate and dynamic realm of liver fibrosis and its complications in the clinical setting.

## AUTHOR CONTRIBUTIONS

**Kostas A. Papavassiliou:** Conceptualization (lead); data curation (lead); writing – original draft (lead). **Athanasios G. Papavassiliou:** Conceptualization (lead); data curation (lead); supervision (lead); writing – review and editing (lead).

## FUNDING INFORMATION

None.

## CONFLICT OF INTEREST STATEMENT

The authors declare no competing financial interests.

## Data Availability

Data sharing not applicable—no new data generated.

## References

[1] Kong M , Zhou J , Kang A , et al. Histone methyltransferase Suv39h1 regulates hepatic stellate cell activation and is targetable in liver fibrosis. Gut. 2024;73(5):810‐825. doi:10.1136/gutjnl-2023-329671 38176898

[2] Kisseleva T , Brenner D . Molecular and cellular mechanisms of liver fibrosis and its regression. Nat Rev Gastroenterol Hepatol. 2021;18(3):151‐166. doi:10.1038/s41575-020-00372-7 33128017

[3] Mederacke I , Hsu CC , Troeger JS , et al. Fate tracing reveals hepatic stellate cells as dominant contributors to liver fibrosis independent of its aetiology. Nat Commun. 2013;4:2823. doi:10.1038/ncomms3823 24264436 PMC4059406

[4] Kisseleva T . The origin of fibrogenic myofibroblasts in fibrotic liver. Hepatology. 2017;65(3):1039‐1043. doi:10.1002/hep.28948 27859502 PMC5476301

[5] Tsuchida T , Friedman SL . Mechanisms of hepatic stellate cell activation. Nat Rev Gastroenterol Hepatol. 2017;14(7):397‐411. doi:10.1038/nrgastro.2017.38 28487545

[6] Snigdha S , Prieto GA , Petrosyan A , et al. H3K9me3 inhibition improves memory, promotes spine formation, and increases BDNF levels in the aged hippocampus. J Neurosci. 2016;36(12):3611‐3622. doi:10.1523/JNEUROSCI.2693-15.2016 27013689 PMC4804016

[7] Lu C , Yang D , Klement JD , et al. SUV39H1 represses the expression of cytotoxic T‐lymphocyte effector genes to promote colon tumor immune evasion. Cancer Immunol Res. 2019;7(3):414‐427. doi:10.1158/2326-6066.CIR-18-0126 30610059 PMC6397681

[8] Lu C , Klement JD , Yang D , et al. SUV39H1 regulates human colon carcinoma apoptosis and cell cycle to promote tumor growth. Cancer Lett. 2020;476:87‐96. doi:10.1016/j.canlet.2020.02.004 32061753 PMC7138631

[9] Jokl E , Mullan AF , Simpson K , et al. PAK1‐dependent mechanotransduction enables myofibroblast nuclear adaptation and chromatin organization during fibrosis. Cell Rep. 2023;42(11):113414. doi:10.1016/j.celrep.2023.113414 37967011

[10] Karamouzis MV , Gorgoulis VG , Papavassiliou AG . Transcription factors and neoplasia: vistas in novel drug design. Clin Cancer Res. 2002;8(5):949‐961. 12006506

[11] Peng D , Fu M , Wang M , Wei Y , Wei X . Targeting TGF‐β signal transduction for fibrosis and cancer therapy. Mol Cancer. 2022;21(1):104. doi:10.1186/s12943-022-01569-x 35461253 PMC9033932

[12] Papavassiliou KA , Basdra EK , Papavassiliou AG . The emerging promise of tumour mechanobiology in cancer treatment. Eur J Cancer. 2023;190:112938. doi:10.1016/j.ejca.2023.112938 37390803

